# Updates on British Thoracic Society Statement on Pleural Disease and Procedures 2023

**DOI:** 10.2174/0118743064286775231128104253

**Published:** 2023-12-01

**Authors:** Ashish Subedi, Prakash Banjade, Sushil Joshi, Munish Sharma, Salim Surani

**Affiliations:** 1 Internal Medicine, Gandaki Medical College, Pokhara, Nepal; 2 Internal Medicine, Manipal College of Medical Sciences, Pokhara, Nepal; 3 Internal Medicine, Nepalese Army Institute of Health Sciences, Kathmandu, Nepal; 4 Sleep Medicine, Baylor Scott & White Health, Dallas, USA; 5 Pulmonary, Critical Care & Pharmacy, Texas A&M University, College Station, TX79016, USA

The British Thoracic Society published the new guidelines for pleural disease in 2023 [1]. The previous guideline was published in 2010 and covered six broad areas- Investigation of unilateral pleural effusion in adults, Management of spontaneous pneumothorax, Management of malignant pleural effusion, Management of pleural infection in adults, Local anesthetic thoracoscopy, and Chest drain insertion and thoracic ultrasound [2]. Pediatric pleural disease, thoracic surgical techniques, and management of bilateral pleural effusions were not covered in the 2010 guidelines. The new evidence-based guideline provides recommendations and good practice points for 1) investigating and managing pleural diseases, including spontaneous pneumothorax, 2) undiagnosed unilateral pleural effusion, 3) pleural infection, and 4) pleural malignancy. Mesothelioma is not included in this guideline as it is already covered in the BTS Guideline for investigating and managing pleural mesothelioma [3]. Benign (non-infectious and non-pneumothorax) pleural diseases and rare pleural diseases are also excluded from consideration. In addition to the main guidelines, a clinical statement on Pleural procedures was released to provide further information [4]. This statement covers technical details of pleural procedures, such as chest tube insertion, indwelling pleural catheter, closed pleural biopsy, and thoracoscopy. It also offers guidance on managing any potential complications.

Regarding Pneumothorax, the 2023 guideline suggests a symptoms-based approach. Conservative treatment is advised if a patient is asymptomatic or experiencing only mild symptoms. For adults with primary spontaneous pneumothorax (PSP), it is recommended to start with ambulatory management as long as they have adequate support and are cared for by well-equipped experts who can provide follow-up care. However, if a patient exhibits high-risk characteristics, such as tension pneumothorax, significant hypoxia, bilateral Pneumothorax, or an underlying lung disease, intercostal drainage (ICD) or needle aspiration (NA) may need to be considered, provided the procedure is safe. NA may lead to a shorter hospital stay than ICD, but the recurrence rate in pneumothorax is similar with both procedures. Notably, the need for future pleural procedures is greater with NA than with ICD.

Graded talc remains the agent of choice instead of ungraded talc, which can be associated with acute respiratory distress syndrome and subsequent mortality [4]. Meta-analyses have shown that chemical pleurodesis has a lower risk of recurrence of PSP and secondary spontaneous pneumothorax (SSP) compared to just using an ICD. Patients treated with thoracic surgery using either VATS or thoracotomy have a lower risk of PSP and SSP recurrence than those treated with an ICD [1].

There is a lack of sufficient data regarding the recurrence of pneumothorax during subsequent pregnancy in patients with a history of pneumothorax. So, there is no clear recommendation for subsequent pregnancies and how to address that. Pregnant women with small asymptomatic pneumothorax can be closely observed. Intervention with ICD or NA is recommended if symptoms arise. Surgical evaluation may be necessary for pregnant women with continuous air leaks due to the risk of potential exacerbation during labor [1].

The 2023 guideline recommends a small-bore chest tube for initial drainage of the infected pleural effusion. Greater than 14F ICD may result in more pain. In 2010, the validity of A RAPID score for pleural infection was not confirmed. However, in 2023, it was validated to determine the mortality risk of patients. Those with higher risks are advised to undergo aggressive treatment. However, there is no evidence that early intervention with tPA/DNAase instillation or surgery (decortication of pleura) benefits patients with high RAPID scores. The MIST 2 trial concluded that in patients with pleural infection, intrapleural t-PA–DNase therapy improved fluid drainage, reduced surgical referrals, and shortened hospital stays [5]. Single-agent TPA or DNAse are not recommended for pleural infection treatment.

When dealing with pleural effusion, it is advisable to undergo USG-guided thoracocentesis. In the case of suspected mesothelioma, which is characterized by poor cytology, an imaging-guided pleural biopsy is recommended [1]. This procedure is typically performed under local anesthesia simultaneously with pleural fluid aspiration or thoracoscopy. Previous guidelines from 2010 advised waiting for cytology reports before proceeding with any intervention.

Thoracoscopic or image-guided pleural biopsy is recommended for undiagnosed pleural pathology, while blind pleural biopsies should be avoided. Awake thoracoscopic pleural and video-assisted thoracoscopic pleural biopsies under general anesthesia have similar diagnostic yields. Both rigid and semi-rigid thoracoscopy can be used to obtain a pleural biopsy. Thoracoscopic pleural biopsy is more likely to provide a definitive diagnosis than image-guided closed pleural biopsy.

When it comes to malignant pleural effusion, there is evidence to suggest that using talc pleurodesis for patients with <25% non-expandable lung can improve their quality of life by reducing chest pain, breathlessness, and recurrence rates of pleural effusion. However, it is important to consider the patient's preference for an Indwelling Pleural Catheter (IPC) or pleurodesis. The question then becomes which modality to use for talc pleurodesis. Both thoracoscopy and talc poudrage pleurodesis, as well as chest drain and talc slurry pleurodesis, aim to induce inflammation and adhesion of the pleural layers to prevent fluid accumulation. Theoretically, thoracoscopy and talc poudrage pleurodesis should allow better coverage of the pleural space due to direct visualization. They may also be associated with a shorter length of stay despite it being a more invasive procedure. The collective evidence from Bhatnagar *et al.* (2019) [6], Walker *et al.* (2016) [7], and Stefani *et al.* (2006) [8] suggest that there is no difference in health-related quality of life, length of hospital stay, chest pain or breathlessness in adults with MPE treated with chest drain and slurry, or thoracoscopy and talc poudrage.

The 2010 guidelines suggested that symptomatic patients benefitting from aspiration with malignant pleural effusion (MPE) should go for talc pleurodesis provided they have expanded non-trapped lungs. Indwelling catheter solution was reserved for those who either failed pleurodesis or had trapped lung initially. The 2023 guideline has recommended indwelling catheters as the first-choice management for MPE. Patients with expansile lungs are provided with an indwelling catheter or talc pleurodesis. For patients with trapped lungs, an indwelling catheter is the choice. However, for those with a limited prognosis, repeat aspiration is suggested.

To summarize, there is no noticeable variation in Pneumothorax recurrence when comparing NA and ICD as the primary management approach for PSP. Conservative and ambulatory management methods play a significant role in handling pneumothorax depending on high-risk characteristics. It may be necessary to take surgical intervention on Pneumothorax related to pregnancy with continuous air leaks before labor to prevent deterioration. The rapid score has been incorporated for risk stratification and aggressive management of pleural infection, which can aid in discussions with patients regarding potential outcomes. Studies indicate no significant difference in outcomes between chest drainage and talc slurry or thoracoscopy and talc poudrage for treating MPE. The use of tPA/DNase to treat pleural infection at a 10:5 ratio for three days, as per the guidelines, instead of waiting for the patient's response and deciding the dose and course, is worth further investigation.

The 2023 pleural disease guidelines by the British Thoracic Society are a remarkable blend of evidence-based advice and expert opinions. These guidelines are built on thoroughly evaluating available evidence, providing a solid evidence base for the recommendations. Additionally, the guidelines have been enriched by the valuable clinical insights of leading specialists in the field. To avoid any biases, the guidelines have been created using a transparent and systematic approach, which involved a diverse panel of experts and rigorous peer review processes.

Table **1** Shows major changes in 2023 guidelines in comparison to previous guidelines.

## Figures and Tables

**Table 1:** Comparison of major changes between older guidelines and 2023 guidelines.

Older guidelines	2023 guidelines
**Pneumothorax** The 2010 guidelines differentiated pneumothorax directly into primary and secondary. **Pleural Infection** A RAPID score was not validated in the 2010 guidelines. The 2010 guidelines state that tPA/DNASE or surgery should be considered after seven days if antibiotic use or chest drain does not lead to improvement in patients. **Pleural Effusion** The 2010 guidelines recommended using ultrasound (USG) to guide procedures like thoracocentesis strictly for safety concerns. **Pleural Malignancy** If mesothelioma is suspected, which has poor cytology, previous guidelines from 2010 suggested waiting for cytology reports before any intervention. 2010 guidelines suggested that symptomatic patients benefitting from aspiration with Malignant pleural effusion (MPE) should go for talc pleurodesis provided they have expanded non-trapped lungs. Indwelling catheter solution was reserved for those who either failed pleurodesis or had trapped lung initially.	2023 guidelines focus on a symptom-led approach for pneumothorax management. The RAPID score has now been validated in 2023, determining patients' mortality risk. The 2023 guidelines suggest assessing patients in 48 hours. The 2023 guidelines recommend that USG should be used to diagnose effusion and suspected malignancy in addition to safety concerns. The 2023 guidelines recommend an imaging-guided pleural biopsy, pleural fluid aspiration or thoracoscopy simultaneously. The 2023 guideline has recommended indwelling catheters as the first-choice management for MPE. Patients with expansile lungs are provided with an indwelling catheter or talc pleurodesis. For patients with trapped lungs, an indwelling catheter is the choice. However, for those with a limited prognosis, repeat aspiration is suggested.
